# Dietary Exposure of the Japanese General Population to Elements: Total Diet Study 2013–2018

**DOI:** 10.14252/foodsafetyfscj.D-22-00003

**Published:** 2022-09-23

**Authors:** Takahiro Watanabe, Yohei Kataoka, Kyoko Hayashi, Rieko Matsuda, Chikako Uneyama

**Affiliations:** 1 Food Safety Information Division, National Institute of Health Sciences, 3-25-26, Tonomachi, Kawasaki-ku, Kawasaki, Kanagawa 210-9501, Japan; 2 Biochemistry Division, National Institute of Health Sciences, 3-25-26, Tonomachi, Kawasaki-ku, Kawasaki, Kanagawa 210-9501, Japan

**Keywords:** total diet study, exposure assessment, Japanese general population, element

## Abstract

Some countries have conducted a total diet study (TDS) focused on the estimation of specific trace elements. Although some results of a Japanese TDS examining trace elements were published, there have been no reports of a nationwide TDS across Japan over a multi-year period to estimate the level of exposure to multiple elements. In the present study, a TDS using a market basket approach was performed to estimate the dietary exposure levels of the general population of Japan to 15 elements, including aluminum (Al), total arsenic (tAs), boron (B), barium (Ba), cadmium (Cd), cobalt (Co), chromium (Cr), total mercury (THg), molybdenum (Mo), nickel (Ni), lead (Pb), antimony (Sb), selenium (Se), tin (Sn), and uranium (U). Samples prepared in eight regions across Japan over a 6-year period were analyzed using validated methods. The robust mean exposure estimates for Al, tAs, B, Ba, Cd, Co, Cr, THg, Mo, Ni, Pb, Sb, Se, Sn, and U were 48, 4.2, 29, 8.6, 0.35, 0.17, 0.49, 0.14, 4.2, 2.8, 0.15, 0.022, 1.8, 0.10, and 0.021 μg/kg body weight/day, respectively. Although the variability in exposure estimates varied greatly from element to element, the relative standard deviations calculated from the robust means and robust standard deviations were ≤ 50% for all elements except Sn. Compared against the health-based guidance values, none of the robust and precise estimates obtained for the target elements would be associated with urgent health risk concern. In addition, the estimated exposure levels were generally in agreement with previously reported estimates, indicating that health risks associated with exposure to these elements have not changed markedly nationwide in Japan in recent years.

## 1. Introduction

Potentially toxic elements are widely present in the environment and also found in foods. Among these elements, trace elements such as arsenic (As), cadmium (Cd), lead (Pb), and mercury (Hg) are the subject of much public concern; these elements have been well-studied due to their high toxicity, and they are regulated by national authorities as contaminants. In contrast, elements such as molybdenum (Mo) and selenium (Se), which are necessary for human life, can have adverse effects on health if ingested in excessive amounts; therefore, a tolerable upper intake level is set for these elements, along with a recommended dietary allowance^1^^)^. Partly because they are not well known, however, concern regarding these elements is not as high as for more toxic elements, resulting in fewer reports of their occurrence in foods or dietary exposure compared with elements of high concern.

Exposure to potentially toxic elements does not necessarily lead to adverse health effects immediately. Whether adverse health effects occur, and if so to what extent, depends on the combination of the toxicity of specific elements and the exposure dose. Therefore, estimation of actual exposure levels is necessary, and these estimates should be compared to health-based guidance values (HBGVs) such as acceptable daily intake (ADI) and tolerable weekly intake (TWI) to assess the degree of human health risk, which is a function of the probability of adverse health effects occurring and the severity of those effects. In the risk analysis paradigm formulated by the Codex Alimentarius Commission in 1994, exposure level estimation is recognized as an essential step for assessing human health risks^2^^,^^3^^,^^4^^)^.

The total diet study (TDS) is useful for estimating dietary intake or exposure levels of a target population to various substances, and such studies have been used to estimate these exposure levels^5^^)^. There are two approaches to performing a TDS: the market basket (MB) approach and the duplicate diet (DD) approach, although the MB approach may be recognized as synonymous with the TDS^6^^)^. In a TDS using a DD approach, duplicate diet samples are prepared by sampling the duplicate diet eaten by the study participants, and exposure levels of targeted substances are estimated based on the results of sample analyses. In a TDS using an MB approach, representative food items consumed by a target population, differentiated by region, etc., are purchased, prepared, and then aggregated into relevant food groups consisting of similar foods. TDS samples prepared in this manner are analyzed by group, and the resulting concentrations are multiplied by a mean daily consumption value to estimate the exposure levels for each food group. The estimated exposures from all food groups are summed to estimate the total exposure, usually referred to as the exposure level. Regulatory authorities in developed countries such as the U.S., Canada, and Australia have conducted TDSs for pesticide residues, hazardous elements, polychlorinated biphenyls, and dioxins and published the results on their web sites. One of the aims of these studies is to confirm that the health risks from ingestion of hazardous substances via the diet is sufficiently low^7^^,^^8^^,^^9^^)^.

A number of countries have conducted TDSs focused on the estimation of specific trace elements, including As, Cd, Pb, and Hg^10^^,^^11^^,^^12^^,^^13^^,^^14^^,^^15^^)^. The results of a TDS examining trace elements including Cd, Pb, and Hg in Tokyo were published on the Tokyo Metropolitan Government web site^16^^)^. Yoshinaga et al. reported the results of a TDS of Pb and 14 trace elements using an MB approach^17^^,^^18^^)^. Hayashi et al. reported the daily exposure level of total As (tAs) and inorganic As (iAs), Pb, and Al of the Japanese population based on the results obtained from a TDS using a DD approach^19^^)^. However, there have been no reports of a nationwide TDS using an MB approach across Japan over a multi-year period to estimate the exposure levels with respect to multiple elements, including elements of both high and low public concern.

To reliably estimate the dietary exposure of the general population of Japan to 15 elements, including elements with high toxicity and others, we performed a TDS using an MB approach over a 6-year period, from 2013 to 2018. Considering the applicability of the validated simultaneous analytical methods, the following 15 elements were selected as targets for estimation: aluminum (Al), tAs, boron (B), barium (Ba), Cd, cobalt (Co), chromium (Cr), total Hg (THg), Mo, nickel (Ni), Pb, antimony (Sb), Se, tin (Sn), and uranium (U). In our study, TDS samples for each of 14 food groups were prepared in eight regions across Japan. A total of 672 samples were analyzed, and 48 exposure estimates were obtained for each element. As a sufficient number of estimates was obtained, algorithm A, an algorithm enabling calculation of robust statistics^20^^)^, was applied to exclude the influence of outliers and calculate the robust means and standard deviations (SDs) of exposure levels. This is the first report of a nationwide TDS across Japan over a multi-year period to estimate robust exposure levels for 15 elements and to compare them with established HBGVs. The estimates obtained in the present study, along with those reported in previous study, provide scientific evidence sufficient to assess the health risks of dietary exposure to these elements.

## 2. Materials and Methods

### 2.1 TDS Samples

**Fig. 1.**
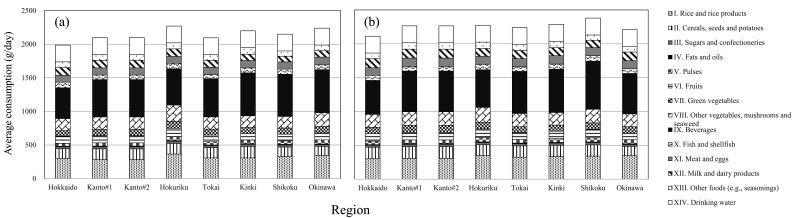
Daily consumption of the 14 food groups in the eight regions of Japan. (a) Daily consumption based on 2008-2010 data used in 2013-2015 TDS. (b) Daily consumption based on 2011-2013 data used in 2016-2018 TDS. Although Kanto#1 and Kanto#2 are the same region in terms of collection of consumption data and had the same value for the average, they were distinguished as different regions in the present study because the TDS samples were prepared in different laboratories sufficiently distant from each other.

TDS samples were prepared annually following the MB approach. Considering increases in the number of food items included and patterns of food consumption, TDS samples were prepared by eight laboratories, mainly local public health institutes in different regions across Japan, from Hokkaido to Okinawa. More than 100 food items were collected from supermarkets by each laboratory and cooked or prepared for consumption in the household manner, such as by boiling and grilling. The items were then aggregated to form a 14–food group composite for the TDS samples according to the food classification used in the National Health and Nutrition Survey in Japan and average daily consumption data (≥1 year of age) for each region obtained by the survey, 2008-2013 (Ministry of Health, Labour and Welfare of Japan). The TDS samples were prepared based on regional average daily consumption data for three consecutive years. Specifically, TDS samples prepared in 2013-2015 were based on average consumption data from 2008-2010 (phase 1), and TDS samples prepared in 2016-2018 were based on average consumption data from 2011-2013 (phase 2). **Fig. 1** shows the average daily consumption of the 14 food groups in the eight regions in phase 1 and phase 2. There were no significant differences in overall consumption pattern between the eight regions through the two phases of our study. The 14 food groups for TDS samples were identical to those previously reported^21^^,^^22^^)^: (I) rice and rice products, (II) cereals, seeds and potatoes, (III) sugars and confectioneries, (IV) fats and oils, (V) pulses, (VI) fruits, (VII) green vegetables, (VIII) other vegetables, mushrooms and seaweed, (IX) beverages, (X) fish and shellfish, (XI) meat and eggs, (XII) milk and dairy products, (XIII) other foods (e.g., seasonings), and (XIV) drinking water. In the actual preparation of the TDS samples, individual food items were arbitrarily selected from among various food items available in supermarkets in each region. After purchase, the items were cooked or prepared and then aggregated into the 13 food groups described above (excluding drinking water). Tap water in each region was collected as food group XIV for drinking water. The prepared TDS samples were kept frozen (−20°C) prior to analysis. In this study, national and international TDS guidelines were taken into consideration for sample preparation and storage^6^^,^^23^^)^.

### 2.2 Element Analyses

All samples were analyzed by a single laboratory of the National Institute of Health Sciences. Except for determination of THg concentration, samples were analyzed by an in-house validated method based on ICP-MS using acidic microwave digestion, as previously reported^24^^)^. Briefly, 0.5 g of each sample was precisely weighed into a tetrafluoromethaxil digestion vessel and subsequently digested with 7 mL of ultrapure nitric acid and 1 mL of hydrogen peroxide using a closed microwave system (ETHOS-One; Milestone, Bergamo, Italy). The digests were quantitatively transferred to 50-mL polypropylene tubes, which were then filled with ultrapure water. The concentrations of 14 elements were determined by ICP-MS (iCAP Q; Thermo Fisher Scientific, Waltham, MA, USA). A solution of mixed internal standards, including beryllium (Be), gallium (Ga), yttrium (Y), indium (In), and thallium (T) was added to all blanks, standards, and sample solutions to correct for non-spectral interference and instrumental drift. To determine THg concentrations, samples were analyzed using an in-house validated method with an Hg analyzer (MA-3000; Nippon Instruments, Tokyo, Japan), as previously reported^25^^)^. The limits of detection (LODs) achieved by the analytical method and used to estimate dietary exposure levels of elements are shown in **Table 1**. The LODs for elements except THg are reproduced from those provided in previous reports^24^^)^.

**Table 1.**
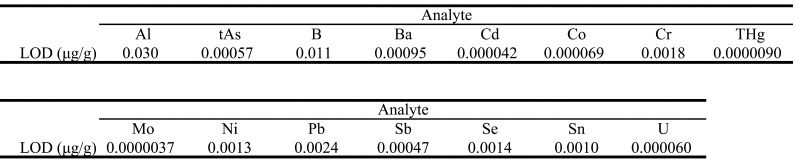
Limit of detection (LOD) estimates for Al, tAs, B, Ba, Cd, Co, Cr, THg, Mo, Ni, Pb, Sb, Se, Sn, and U.

The estimated LODs were 3-fold the standard deviation of the quantitative values obtained in an experiment conducted on the operation blank with the same apparatus and equipment used in these analyses.

### 2.3 Analytical Quality Control

Analytical quality control is important to obtain reliable exposure estimates. The performance of multi-element analytical methods used in the present study was evaluated based on the results of 5 repeated analyses of fortified samples prepared using 14 groups of control samples developed to match the TDS samples and matrixes of each food group. The fortified levels were determined by considering the actual concentrations detected in the TDS samples. Estimates of trueness and repeatability for 14 elements obtained from a previous study^24^^)^ are summarized in **Table 2**. For almost all elements, the trueness was in the range 80-120%, and the repeatability (relative standard deviation; RSD) was less than 20%. The estimates of trueness and repeatability for 196 analyses, representing the total number of combinations of 14 elements and 14 food groups, were compared with the criteria given in the Codex procedural manual^26^^)^. Although 12 estimates did not meet the trueness criterion and 1 estimate did not meet the repeatability criterion, all estimates were considered sufficient to analyze the TDS samples, indicating the validity of this analytical method. Quality assurance through analysis of certified reference materials (CRMs) should be subject of future research. Based on the results of 10 repeated analyses of CRM 7402-a supplied by the National Metrology Institute of Japan, the trueness of the THg analytical method was estimated at approximately 95%, and the estimated intra-laboratory reproducibility was less than 2.5%, indicating that this analytical method was valid^25^^)^. Through all analyses, the linearity of the calibration curve was confirmed as appropriate for each measurement (R^2^>0.999), and no unexpected signals were detected in the blank controls.

**Table 2.**
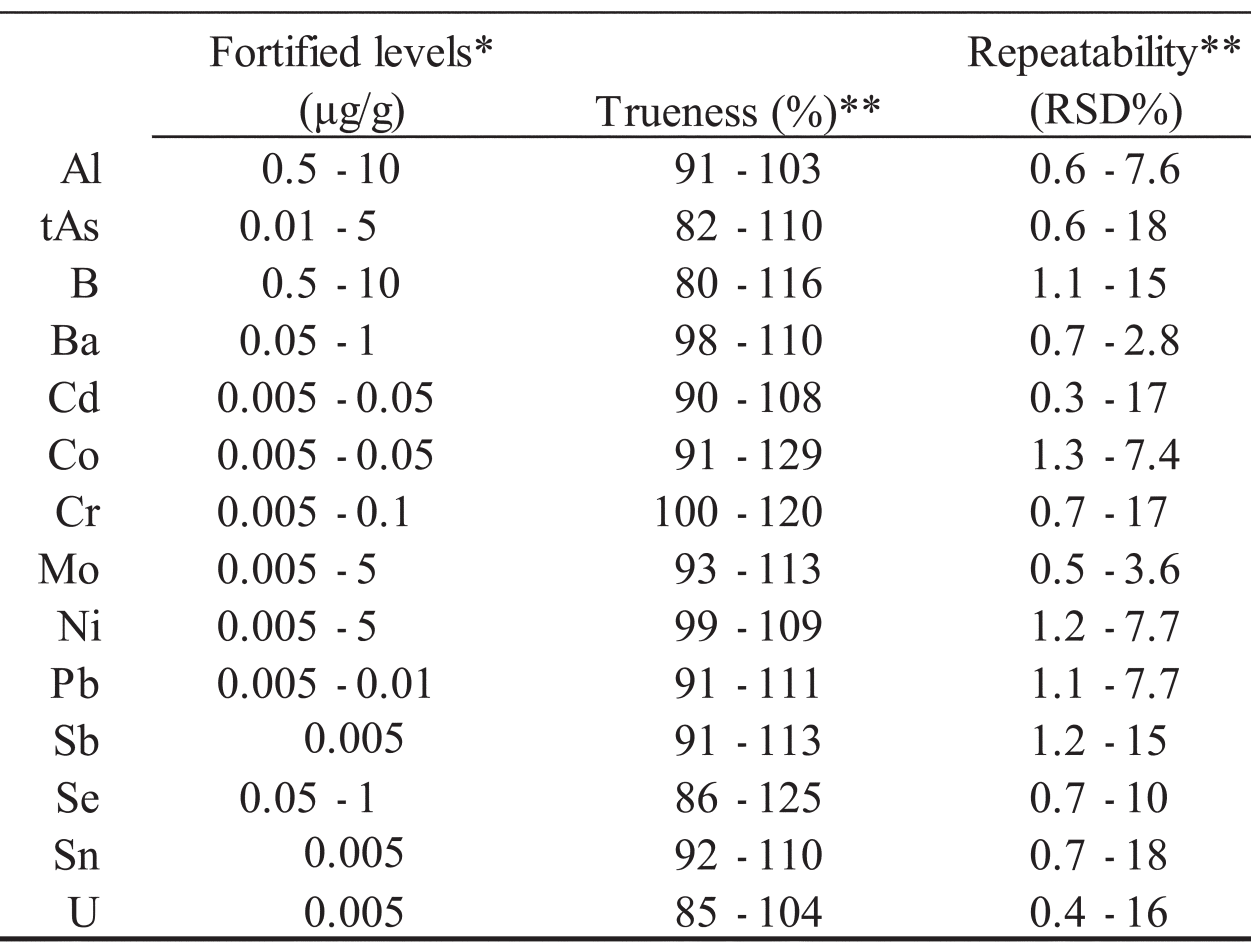
Trueness and repeatability of the multi-element analytical method used in the present study.

Estimates of trueness and repeatability for 14 elements obtained from a previous study^24^^)^ are summarized. Trueness and repeatability were estimated based on the repeated analysis (n=5, 980 total) of fortified samples prepared using 14 groups of control samples developed to match the TDS samples and matrixes of each food group.

*Fortified level depending on the actual concentration detected in the TDS samples.

**Minimum and maximum estimates obtained from analyses of 14 food groups are shown.

### 2.4 Estimation of Dietary Exposure Level

TDS samples were analyzed to determine the concentrations (μg/g) of each element. To estimate daily dietary exposure to each element (μg/person/day) from the TDS samples, the concentrations determined for each element were multiplied by a weight (g) equivalent to the average daily consumption by Japanese of the food items included in the TDS samples. The estimated daily exposure to an element during a given year in a given region was considered the sum of the daily exposure to the element obtained from analyzing the TDS samples of food groups I through XIV prepared in that region in that year. However, the 2013 THg exposure alone was estimated based on the results of analyses of TDS samples of food groups X (fish and shellfish) and XI (meat and eggs), which were shown to contain high levels of Hg in a preliminary analysis.

As the data from the National Health and Nutrition Survey include no information on food consumption per unit body weight, we first estimated daily exposure per person as described above. However, it is also necessary to perform comparisons against HBGVs in exposure assessments; thus, daily exposure per unit body weight (μg/kg bw/day) was estimated by dividing the daily exposure per person by the mean body weight. The mean body weight among Japanese of all age groups (≥1 year of age) was assumed to be 50 kg. Although the present mean body weight exceeds 50 kg according to statistical data, consistency with previous studies was given priority in the present study.

The analytical values were handled as follows. When the result of an analysis of an element was below the LOD, the lower and upper bounds of the daily exposure for the element were estimated by performing two types of calculations: one assuming the concentration of the element was zero, and the other assuming the concentration was LOD/2.

### 2.5 Robust Statistics

Robust values of the mean and SD of the data for daily elemental exposure were calculated following Algorithm A provided in Annex C of ISO 13528^20^^)^.

(1) Calculate initial values for x^*^ and s^*^ as:

x^* ^=
*median ofx_i
 _***(***i *=
1,2,…***p*** )

s^*
^= 1.483 *median of* |*x_i
_*−
x^*^|*_ _***(***i
*=
1,2,…***p*** )

(2) Update the value of x^*^ and s^*^ as follows. Calculate:

δ =
1.5s^*^

For each *x_i _*(*i *= 1,2,…*p*), calculate

x^* ^−δ, if
*x_i_* < x^*^
− δ

$${x_{i}}^{*}\;=\;x^{*}+\delta$$

*x_i_*, otherwise

(3) Calculate the new value of $x^{*}$ and $s^{*}$ from:

$$x^{*}=\frac{\sum x_{i}^{*}}{n}$$

$$s^{*}=1.134\sqrt{\frac{\sum {\left(x_{i}^{*}-x^{*}\right)}^{2}}{n-1}}$$

(4) The robust estimates x^*^ and s^*^ may be derived by an iterative calculation (i.e. by updating the values of x^*^ and s^*^ several times using the modified data, until the process converges).

## 3. Results and Discussion

### 3.1 Analytical Results of TDS Samples

TDS samples (n=672) prepared between 2013 and 2018 were analyzed. The detection rates for the elements in each TDS sample and their concentrations, if detected, are presented. As shown in **Table 3****,** the detection rates varied greatly depending on the element. For instance, tAs and B were detected in nearly 100% of all food groups except group XIV (drinking water). Sn, on the other hand, was detected at a rate of more than 50% in a lower number of specific food groups. Moreover, elements such as THg occurring in food group IV (fats and oils) were detected rarely (detected in only 1 of 40 samples). As shown in **Table 4**, concentrations of detected elements varied widely even within a specific food group and also varied widely among food groups. Several elements, including those that are subject to regulation as hazardous substances, such as As, Cd, Hg, and Pb, were present at very low concentrations, but we were able to detect and quantify those elements using an analytical method with a high detection capability (i.e., with a low LOD).

**Table 3.**
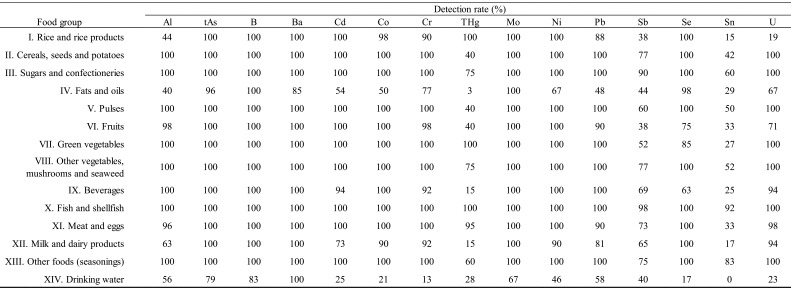
Detection rates of elements by food group.

For all elements except THg, the rates shown are the proportion of samples in which the respective elements were detected in 48 samples analyzed by food group. The THg exposure in 2013 was estimated by analyzing only samples of food groups X and XI. Therefore, the detection rates shown for THg are the proportion of samples in which THg was detected among 40 samples of the respective food groups, except groups X and XI.

**Table 4a.**
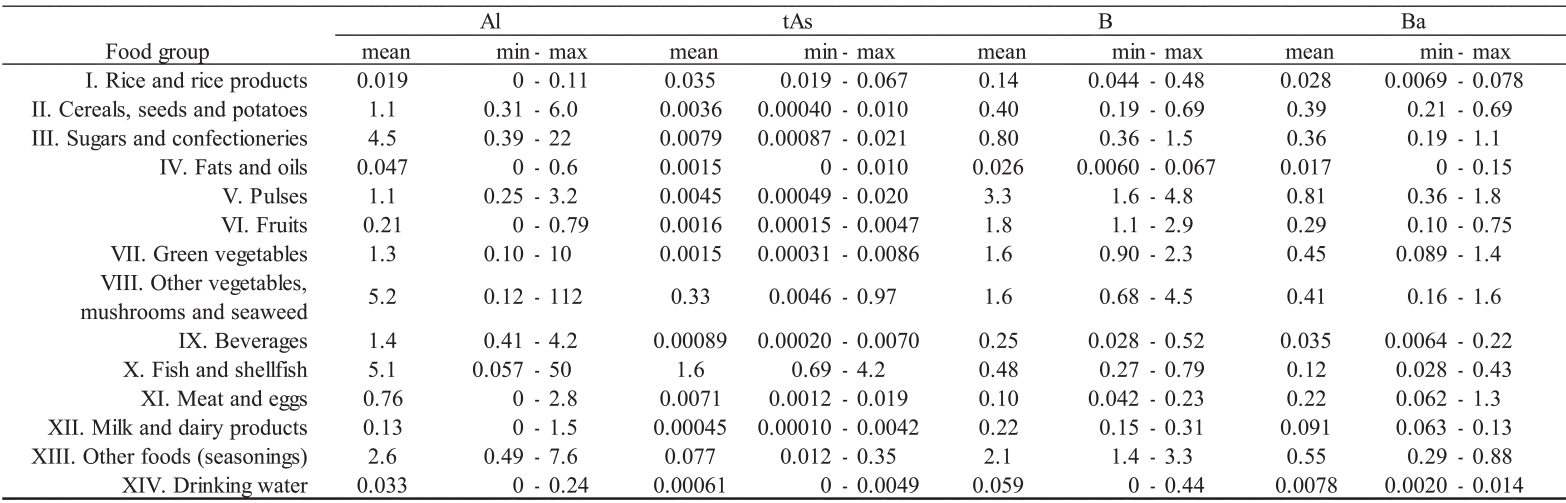
Concentrations of elements (Al, tAs, B, Ba) by food group (μg/g).

The number of samples analyzed for each combination of elements and food groups was 48 (total number was 672 for each specific element).

Analytical results below the LOD are shown as zero; otherwise, they are shown to two significant figures, where possible.

**Table 4b.**
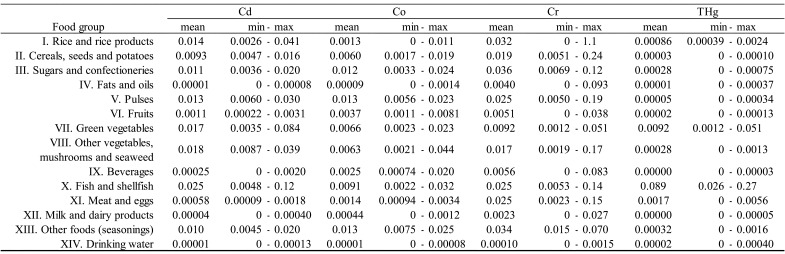
Concentrations of elements (Cd, Co, Cr, THg) by food group (μg/g).

The number of samples analyzed for each combination of elements and food groups was 48 (total number was 672 for each specific element).

Analytical results below the LOD are shown as zero; otherwise, they are shown to two significant figures, where possible.

**Table 4c.**
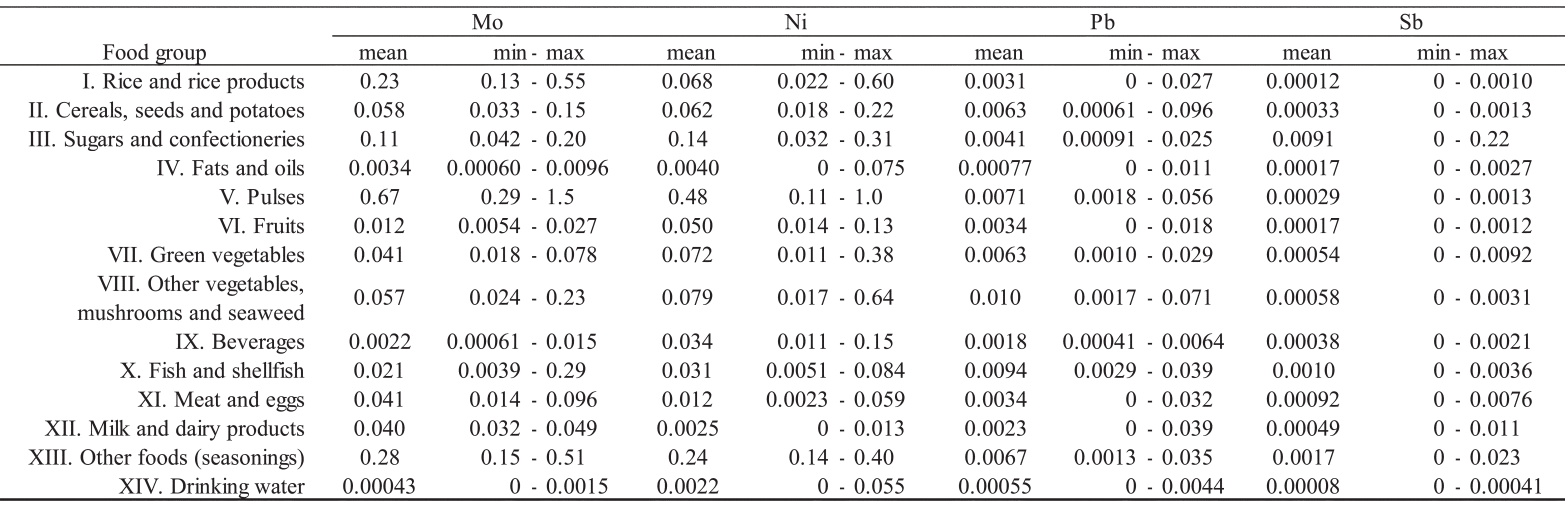
Concentrations of elements (Mo, Ni, Pb, Sb) by food group (μg/g).

The number of samples analyzed for each combination of elements and food groups was 48 (total number was 672 for each specific element).

Analytical results below the LOD are shown as zero; otherwise, they are shown to two significant figures, where possible.

**Table 4d.**
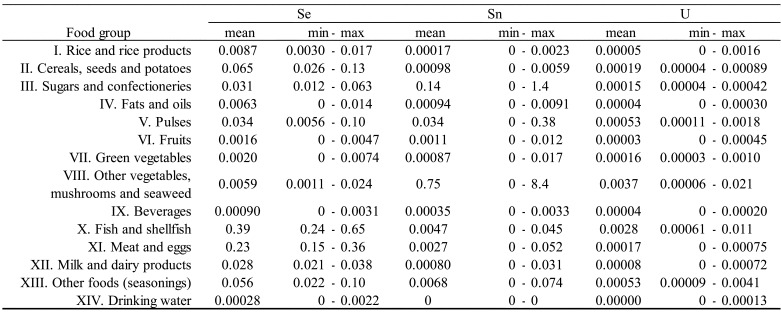
Concentrations of elements (Se, Sn, U) by food group (μg/g).

The number of samples analyzed for each combination of elements and food groups was 48 (total number was 672 for each specific element).

Analytical results below the LOD are shown as zero; otherwise, they are shown to two significant figures, where possible.

### 3.2 Estimation of Dietary Exposure Levels

#### 3.2.1 Initial exposure estimates

**Table 5** shows the ranges and means (along with SDs and RSDs) of estimates (n=48) for exposure levels of each element. The data shown also include the upper and lower bounds of an estimate in cases where an element was not detected (ND) in the analysis, that is, cases in which the concentration was below the LOD. The difference between the mean upper bound and the mean lower bound was zero for tAs, B, Ba, Cd, Co, THg, Mo, Ni, Se, and U and 18%, even at its greatest, for Sb. Owing to the use of an analytical method that functionally determined the concentrations of each element contained in the actual TDS samples and was confirmed to have satisfactorily low LODs^24^^)^, the frequency of analytical results of ND was low, and the values of LOD/2 used in estimating the upper bounds for these results were sufficiently low. Given the small differences between the upper and lower bounds of exposure estimates, the statistical analysis results shown hereafter will indicate those of the upper bound estimates, in order to be conservative in terms of risk analysis. Nevertheless, the statistical analysis results regarding the lower bound estimates were practically the same as for upper bound estimates.

**Table 5.**
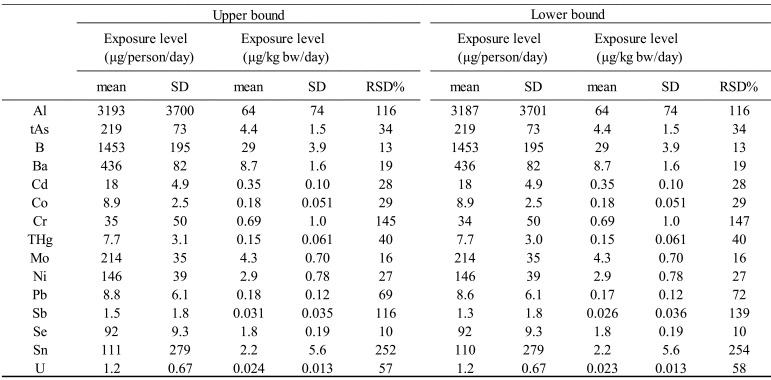
Normal statistics for dietary exposure level of the Japanese population to 15 elements, including As, Cd, Pb, and Hg.

The abbreviation "tAs" represents total arsenic, and "THg" represents total mercury.

Statistics were calculated based on 48 estimates for each element.

**Fig. 2a.**
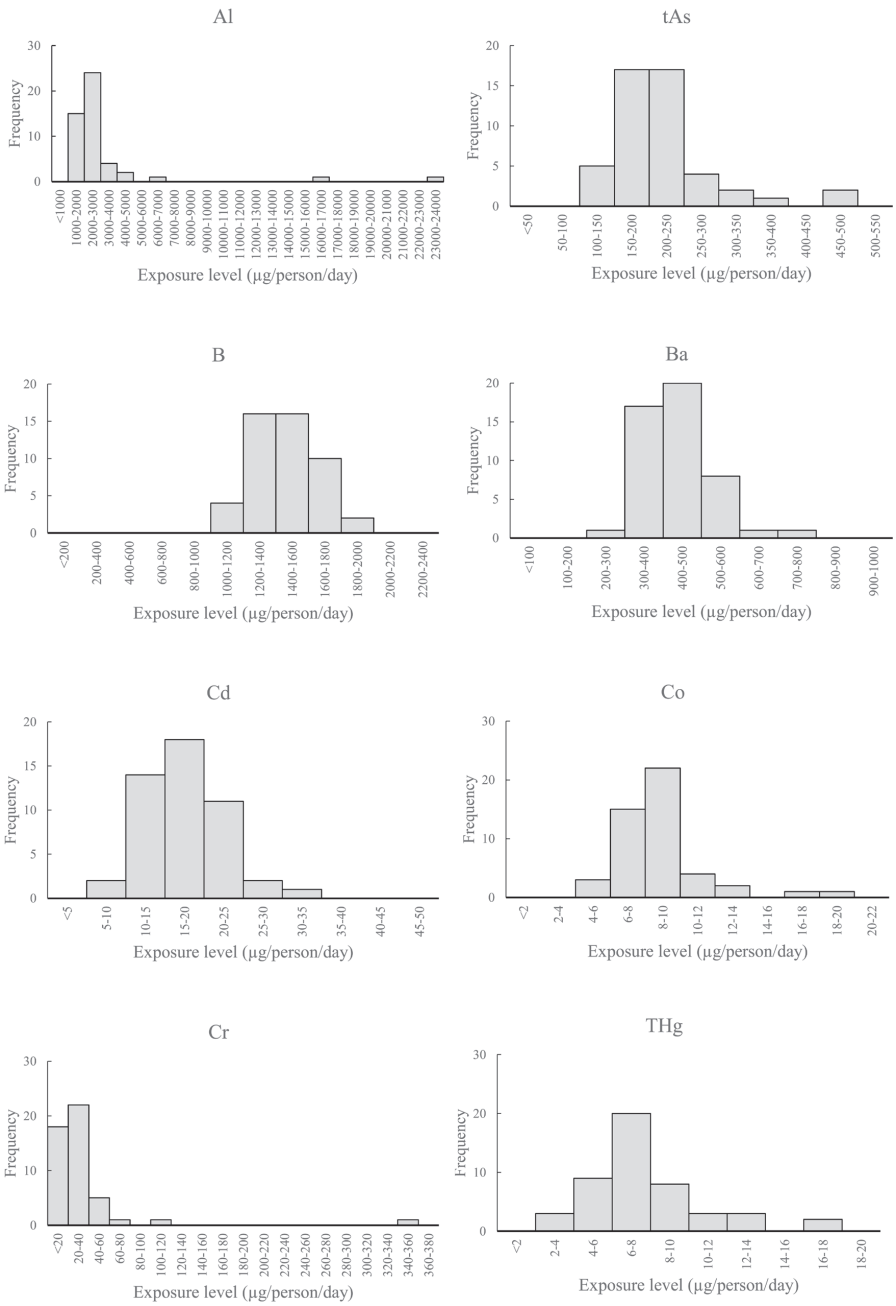
Histograms of exposure estimates for Al, tAs, B, Ba, Cd, Co, Cr, and THg in eight regions of Japan over the 6-year study period (n=48, each element).

The RSDs of exposures varied greatly from element to element, being the smallest at 10% for Se and the greatest at 250% for Sn. The RSD was 20% or less for B, Ba, and Mo, and Se; between 20% and 70% for tAs, Cd, Co, THg, Ni, Pb, and U; and 100% or greater for Al, Cr, Sb, and Sn.

Fig. 2 shows the histograms of exposure estimates for each element over the 6-year study period for the eight regions across Japan. The histograms of exposure estimates for B, Ba, Mo, and Se, for which the RSD was not more than 20%, are in the shape of a near symmetric peak with the top in the center and reveal no substantial outliers. The histograms of exposure estimates for tAs, Cd, Co, THg, Ni, Pb, and U, for which the RSD was between 20% and 70%, show most of the values within the peak near the center and several outliers on the high-exposure side. These outliers away from the center are believed to have contributed to the higher SD. The histograms of exposure estimates for Al, Cr, Sb, and Sn, for which the RSD was 100% or greater, show a peak formed on the low-exposure side by a majority of the exposure estimates, which extends to the high-exposure side due to the presence of several exposure estimates higher than the estimates forming the peak, including one or two high estimates that are several times the maximum estimate of the peak.

**Fig. 2b.**
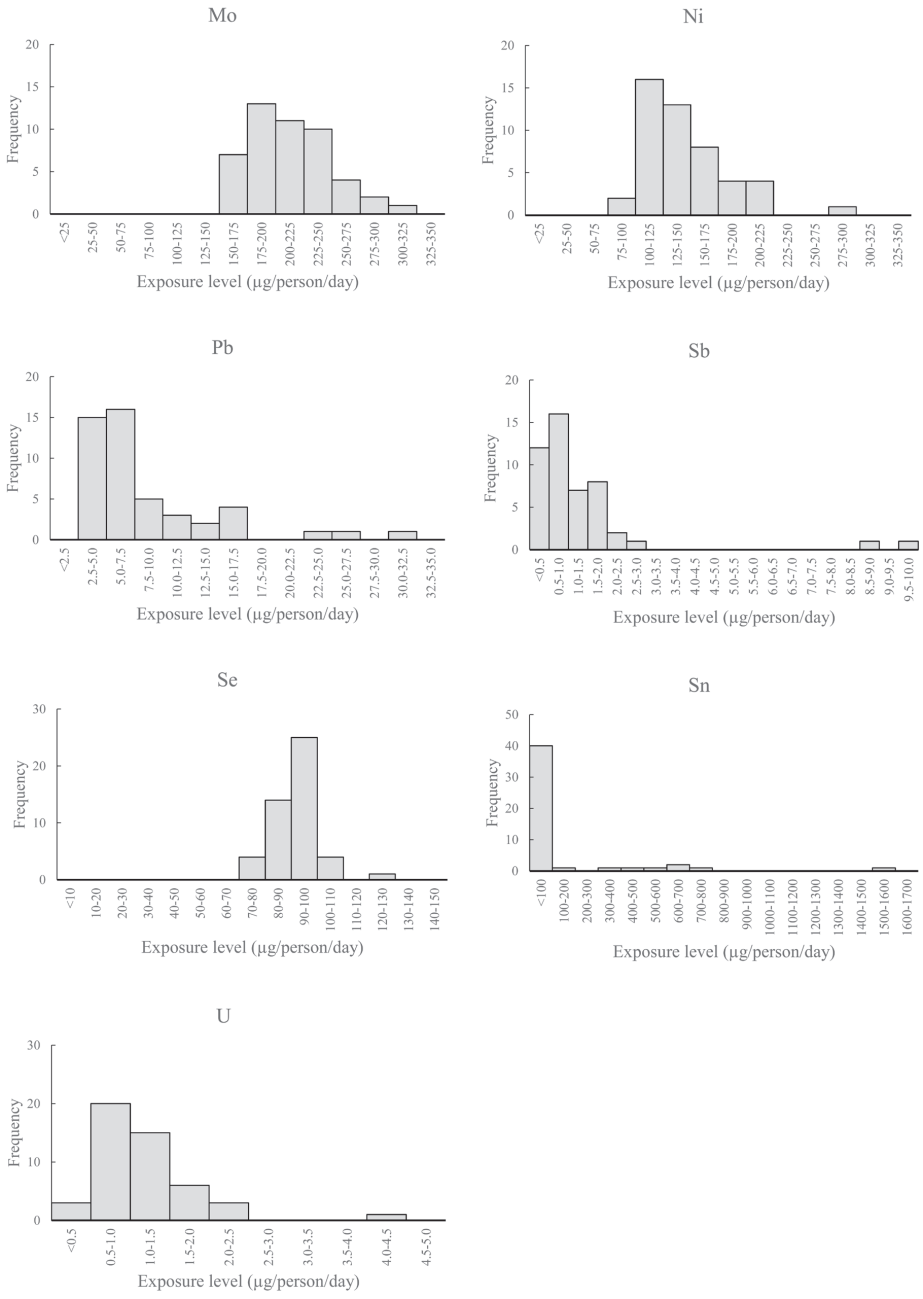
Histograms of exposure estimates for Mo, Ni, Pb, Sb, Se, Sn, and U in eight regions of Japan over the 6-year study period (n=48, each element).

#### 3.2.2 Robust estimates of exposure

As shown in the histograms presented in **Fig. 2**, for some of the elements, the exposure estimates varied substantially from the rest of the estimates; thus, an attempt was made to calculate robust means and robust SDs, which are less susceptible to the effect of these so-called outlier estimates, along with the calculation of RSDs based on such robust statistics (**Table 6**). Except for Sn, the RSDs calculated from the robust means and robust SDs were less than 50%. The TDS samples prepared by the MB approach consisted of a mixture of roughly 100 food items. These food items were prepared at ratios designed to reflect the average amount of each food item consumed by the public. With regard to the combination of elements and food items, some elements may be distributed unevenly in certain food items, such as in the case of Hg, which is present primarily in fish as methyl Hg (MeHg). Nevertheless, despite the impact of the uneven distribution of such elements on variability, the variabilities of the exposure estimates obtained in the present study were relatively small, at about 50% in terms of RSD, partly owing to the effect of using robust statistics.

**Table 6.**
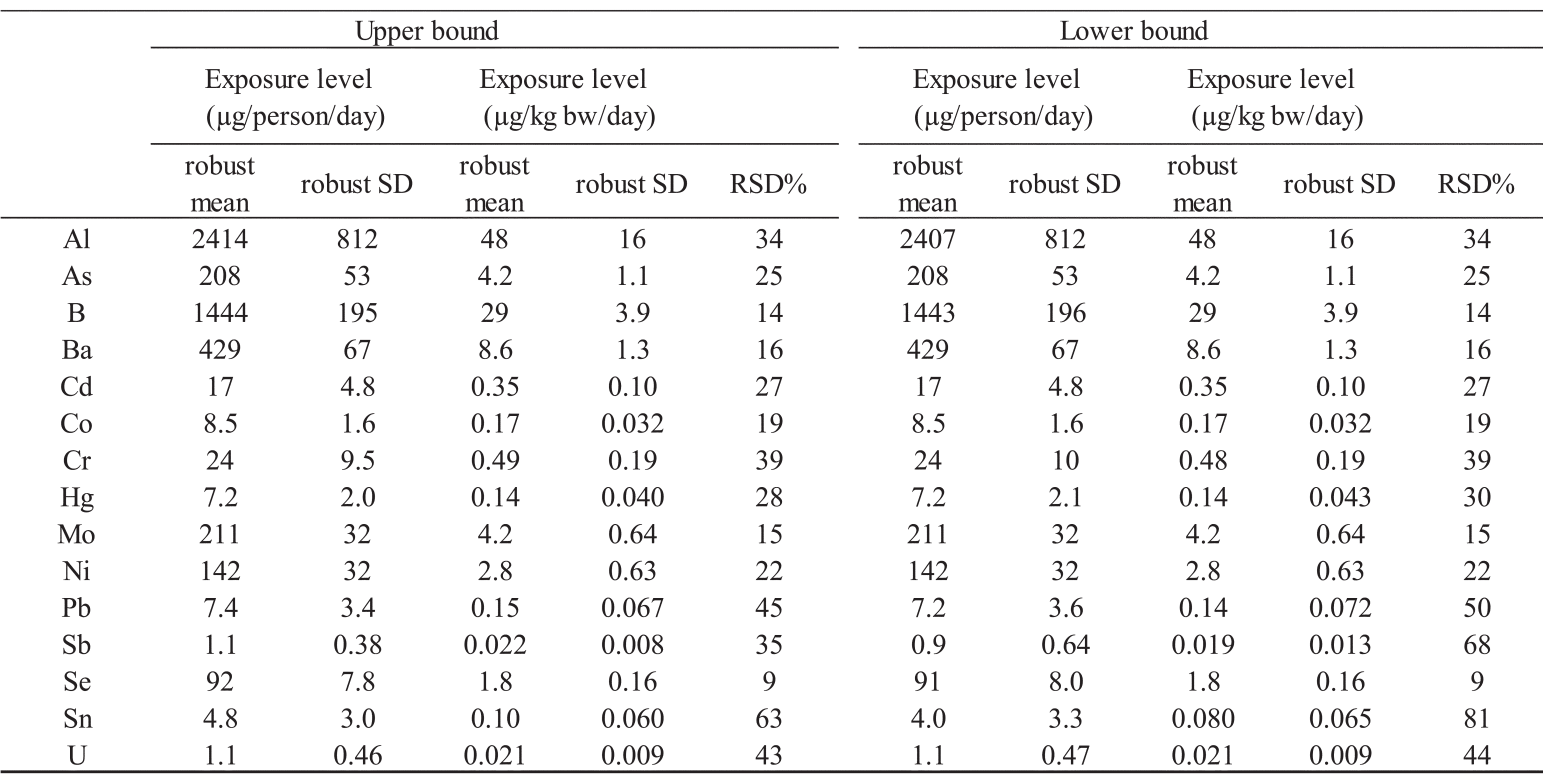
Robust statistics-based exposure estimates.

The abbreviation "tAs" represents total arsenic; and "THg" represents total mercury.

Statistics were calculated based on 48 estimates for each element.

The histogram of Sn exposure estimates (Fig. 2b), for which the RSD calculated using robust statistics was 63%, exhibited a unique pattern in which 40 of the 48 estimates were <100 µg/person/day, and the remaining eight estimates were scattered over the range 100 to 1600 µg/person/day. As such, numerous Sn estimates could be considered outliers. The inability to exclude adequately the impact of such outliers in the calculation of robust statistics is believed to have caused the large RSD in the calculation.

For the exposure estimates for B, Ba, Mo, and Se, the RSDs calculated using normal statistics and those calculated from robust statistics were not more than 20%, with little difference observed. For a distribution that can be considered normal, the 95% confidence interval of its population mean is given by the following equation; $population\;mean=sample\;mean\;\pm 1.96sample\;SD/\sqrt{n}\;$ (*n* indicates number of samples). Therefore, from the results described above, the confidence intervals of the 48 mean exposure estimates for these four elements are believed to be plus or minus several percent. For the exposure estimates for tAs, Cd, Co, THg, Ni, Pb, and U, for which the normal RSDs were 20% to 70%, indicating somewhat large fluctuations, the RSDs calculated from robust statistics were smaller, in the range of 20% to 50%. From these results, the confidence intervals of the 48 mean exposure estimates for these seven elements are believed to be within ±10%.

### 3.3 Time Profiles of Exposure Estimates and Inter-region Variability

The 2013 to 2018 time profiles of the mean exposure estimates for each element and their SDs are shown in **Fig. 3**. The mean exposure estimates for B, Ba, Mo, and Se varied little from year to year, and their SDs, which describe the variability among regions, were also small. The exposure estimates for Cd showed a slightly high variability among the regions but fluctuated little from year to year. Compared with the exposure estimates for B, Ba, Mo, and Se, the mean exposure estimates for tAs, Co, THg, Ni, Pb, and U were not different in terms of annual fluctuation but showed a greater inter-region variability depending on the year of estimation. In particular, a large variability in the exposure estimates for Pb was observed among the regions. The mean exposure estimates for Al, Cr, Sb, and Sn varied greatly between certain years but showed no increase or decrease over the 6 years for which the estimates were made, indicating that the exposure estimate happened to be high in certain years. The respective inter-region variabilities in exposure estimates for each of these five elements were very large in certain years. The years when the mean was high closely coincided with the years when the SD was large, and the means and SDs were large in the years in which the outlier estimates on the high-estimate side were observed, as seen in the histograms shown in **Fig. 2**.

**Fig. 3a.**
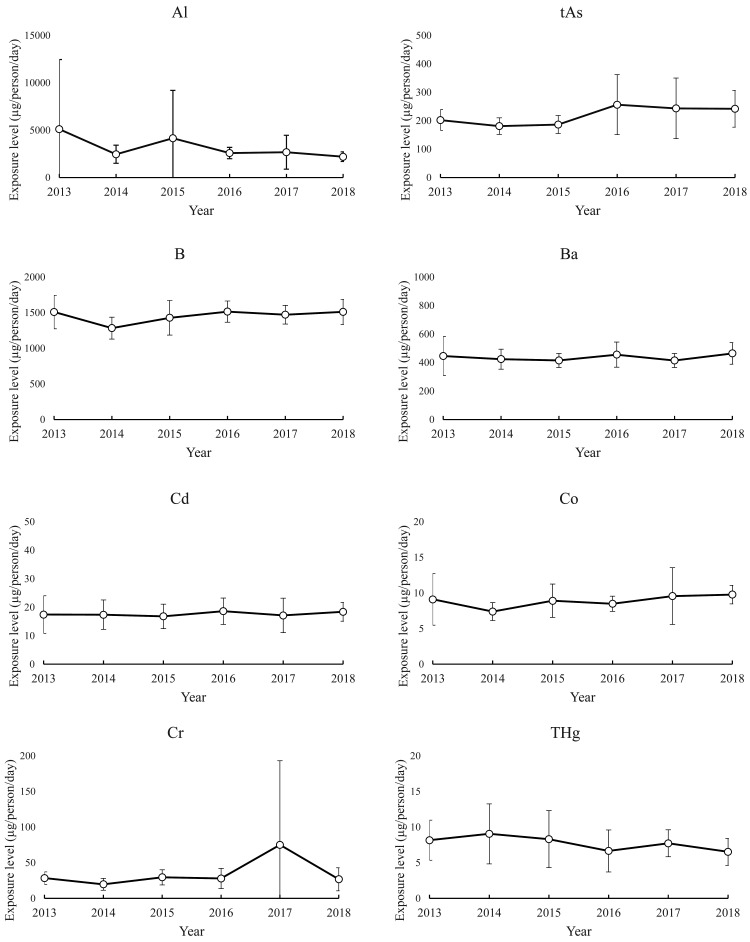
Inter-regional variation and 6-year time profiles of annual exposure estimates (Al, tAs, B, Ba, Cd, Co, Cr, and THg) (n=8, each year).

**Fig. 3b.**
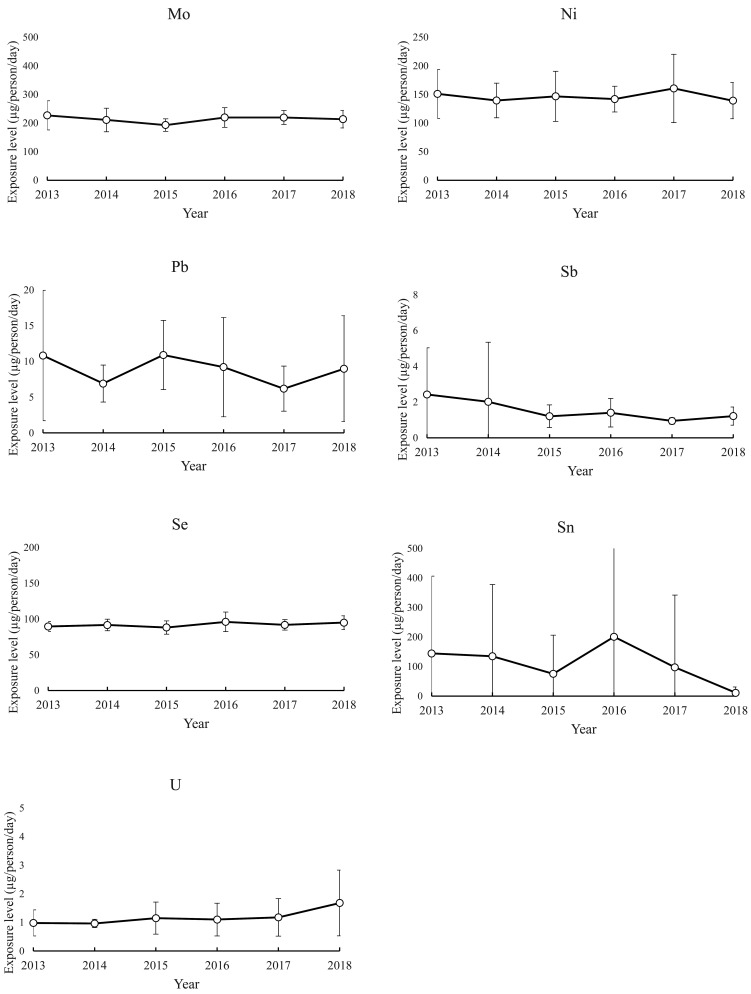
Inter-regional variation and 6-year time profiles of annual exposure estimates (Mo, Ni, Pb, Sb, Se, Sn, and U) (n=8, each year).

### 3.4 Elements with Large Variability in Exposure Estimates and Factors Explaining Such Variability

The exposure estimates for Al, Cr, Sb, and Sn varied greatly, with RSDs of 100% or more as calculated using normal statistics. For these four elements, extremely high exposure estimates in certain years in certain regions increased the variability in the overall 48 estimates over the 6-year period.

The mean exposure estimate for Al over the 6-year period and the SD based on the analytical results of the TDS samples prepared in one particular region (referred to as City A) were both greater than those in the other regions. The exposure estimates in City A were high in certain specific years but not high throughout all 6 years. A detailed analysis of the exposure estimates for Al in City A by food group showed very high exposures from food group VIII in both 2013 and 2015, and the large amount of Aonori (green laver) included in the TDS samples of food group VIII. Berik et al. reported that Al concentrations in green laver are as high as Fe concentrations^27^^)^. Although the actual concentration in the green laver product was unknown, the inclusion of large amounts of the product in TDS samples was thought to be responsible for the high Al exposure level. A by-region comparison of mean exposure estimates for Sn over the 6-year study period showed that the exposure estimates in particular regions (referred to as City B and City C) were far greater than in others, and a by-food-group comparison of exposures showed a substantial contribution from food group VIII. Food group VIII included light-colored vegetables and mushrooms, in addition to seaweed. Bamboo shoots and boiled bamboo shoots were common food items included in the TDS samples of food group VIII prepared in City B and City C but not in the TDS samples prepared in the rest of the regions. Sn is known to leach from some cans used in producing or transporting boiled bamboo shoots^28^^)^. Thus, the Sn that leached from the cans into the boiled bamboo shoots may have caused the high exposure estimates.

The large variation in the exposure estimates for Cr and Sb were considered to be due to contamination rather than to specific foods^29^^,^^30^^)^. As mentioned by Yoshinaga et al. in their study of Pb exposure estimates, it is necessary to continue to be mindful of target element contamination from the equipment and apparatus used in preparing TDS samples and from the analytical environment^17^^)^.

### 3.5 Exposure Assessment

**Fig. 4.**
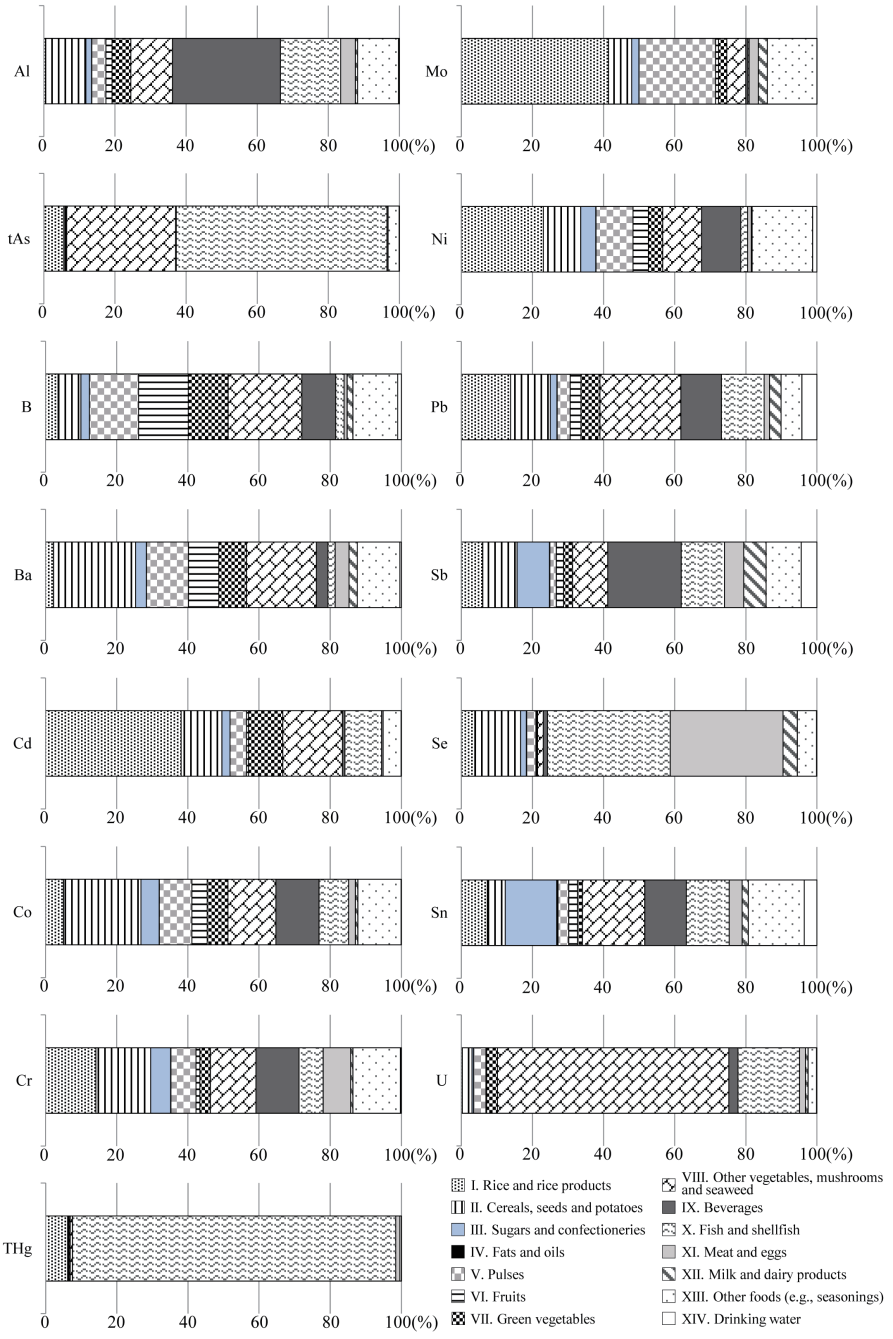
Contribution of food groups to total dietary exposure levels of elements across the entire Japanese population in 2018. The contribution (%) of each food group to total exposure was calculated based on the TDS sample from eight regions.

The tolerable daily intake (TDI) established by the Food Safety Commission of Japan (FSCJ) for each element and the daily exposure to each element estimated in the present study as the robust mean are shown **Table 7**. Given that TWI levels have been established for Al, Cd, and MeHg, for convenience, these values were converted to per day values. The contribution of each food group to the total exposure calculated for each element is shown in **Fig. 4**.

**Table 7.**
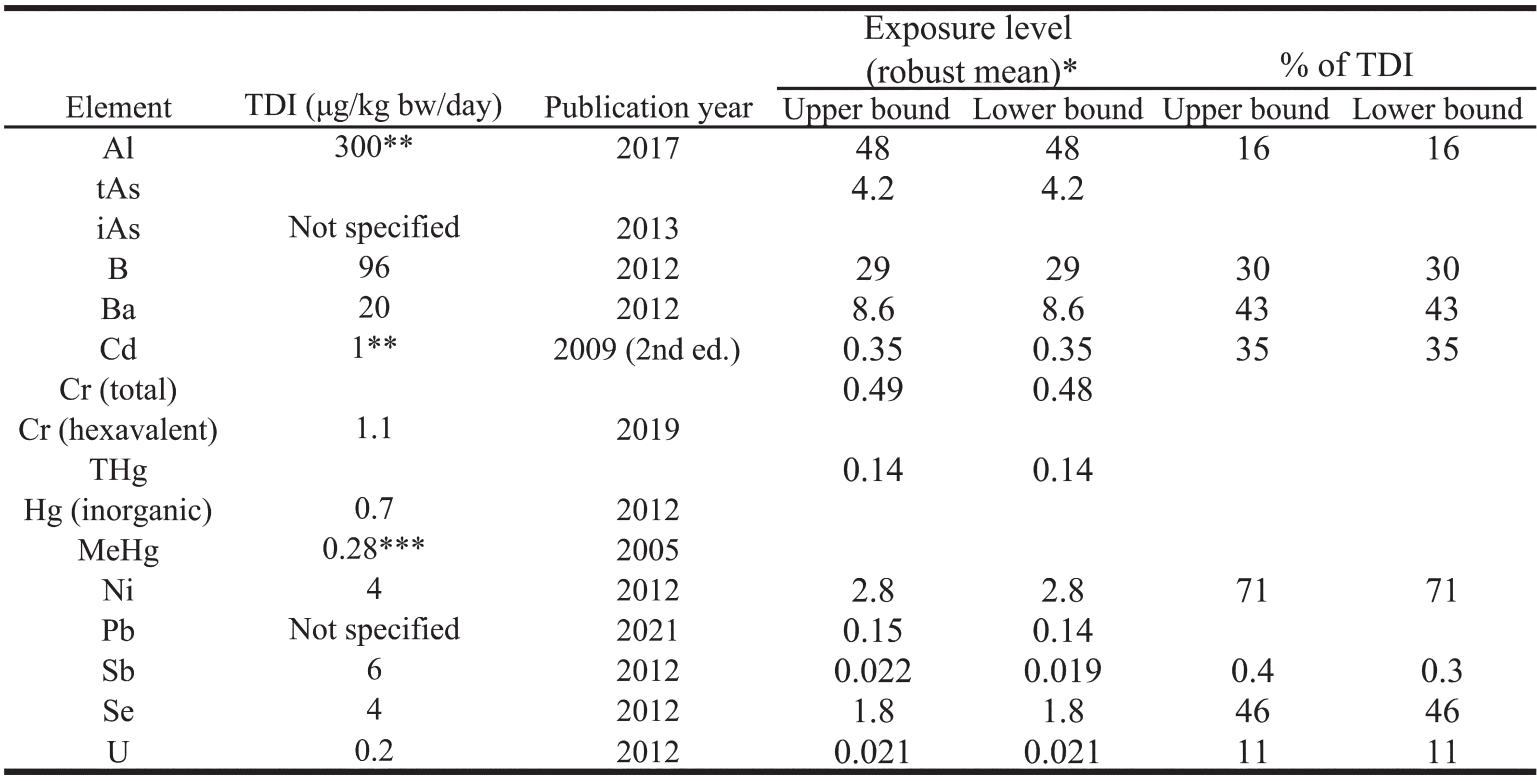
Comparison of exposure estimates and TDI levels established by the Food Safety Commission of Japan.

The abbreviation "iAs" represents inorganic arsenic.

*Units for exposure level are in accordance with those for TDI.

**Value was converted from TWI level.

***Value was converted from TWI level established for women who are pregnant or may become pregnant.

The following discussion is provided for each element. The FSCJ risk assessment report for each element referenced in the following discussion is available on the FSCJ website^31^^)^.

**Al**

The robust mean of Al exposure estimated in the present study was 48 μg/kg bw/day, with a range of 32-61 μg/kg bw/day. The ratio of Al exposure to TWI was 16%. Food group IX (beverages) contributed the greatest amount (30%) to total exposure to Al. The Al exposure level estimated in the present study was consistent with the estimate (45.4 μg/kg bw/day) reported by Hayashi et al. in a TDS using the DD approach^19^^)^. This level was not considered to be of concern for the general population, although caution should be exercised for certain high-exposure groups, as it is known that infants and toddlers are exposed to higher amounts of Al per body weight than adults.

**As**

The robust mean of tAs exposure was 4.2 μg/kg bw/day, with a range of 3.1-5.2 μg/kg bw/day. The contribution to tAs exposure was highest in food group X (fish and shellfish), at 59%, followed by 31% in food group VIII (other foods, e.g., seasonings). A similar tAs exposure level (2.31 μg/kg bw/day) was reported by a previous study^19^^)^.

Although the FSCJ concluded in 2013 that it is not possible to determine whether there is a threshold above which iAs has no carcinogenic effect, it is possible that some Japanese people ingest iAs at levels of several μg/kg bw/day, in excess of the no observable adverse effect level (NOAEL) or benchmark dose lower confidence limit (BMDL), which were derived based on various assumptions, and further research is required. The robust mean of tAs estimated in the present study was similar to the NOAEL or BMDL estimated by the FSCJ in its iAs assessment report. However, the contribution to tAs exposure was highest in food groups X and VIII (i.e., fish and seaweed), and seafood has been found to be a major source of organic As. Assuming that 90% of tAs exposure is from seafood and that iAs in seafood represents 10% of tAs, iAs exposure is an order of magnitude lower than the reference value. Although iAs is certainly a hazardous contaminant that should be controlled, as shown in Fig. 3a, tAs exposure has remained stable at a certain level, suggesting that no extreme changes in health risk have occurred.

**B**

The robust mean of B exposure was 29 μg/kg bw/day, with a range of 25-33 μg/kg bw/day. Only the contribution of food group VIII to total B exposure exceeded 20%.

The ratio of B exposure to TDI was 30%, with no foods being a particularly significant source of B. Thus, B does not seem to be of particular concern.

**Ba**

The robust mean of Ba exposure was 8.6 μg/kg bw/day, with a range of 7.3-9.9 μg/kg bw/day. Food group II (cereals, seeds, and potatoes) contributed the greatest amount (23%) to total Ba exposure. The Ba exposure level estimated in the present study was consistent with the previously reported estimate of 8.1 μg/kg bw/day^18^^)^.

The ratio of Ba exposure to TDI was 43%, which at first consideration appears to indicate high occupancy. However, the TDI for Ba was derived using an uncertainty factor of 10 for the non-toxic dose based on epidemiologic studies of blood pressure and medical history in local residents drinking water with high Ba concentrations. Therefore, there should be minimal concern for serious health effects even with TDI-equivalent exposures.

**Cd**

The robust mean of Cd exposure was 0.35 μg/kg bw/day, with a range of 0.25-0.45 μg/kg bw/day. The ratio of Cd exposure to TWI was 35%. The contribution to total Cd exposure was highest in food group I (rice and rice products), at 38%, followed by 17% in food group VII and 11% in food group II. The Cd exposure level estimated in the present study was consistent with the estimate (0.35 μg/kg bw/day) reported by the Tokyo metropolitan government in 2020^32^^)^.

These results remained essentially unchanged from the 2008 FSCJ reported estimated intake of 2.8 μg/kg bw/week (0.4 μg/kg bw/day), with 37.2% derived from rice, 16.6% from vegetables and seaweeds, 16.1% from seafood, 12.9% from cereals and potatoes, and 17.2% from other sources.

**Cr**

The robust mean of Cr exposure was 0.49 μg/kg bw/day, with a range of 0.30-0.68 μg/kg bw/day. No food group contributed more than 20% to total Cr exposure, with the highest contribution from food groups I and II, at almost 15%. A slightly higher Cr exposure level of 1.1 μg/kg bw/day has been reported^18^^)^.

In 2019, the FSCJ established a TDI of 1.1 µg/kg bw/day for hexavalent Cr. This assessment was to set the TDI for the toxic chemical form of Cr that can be present in drinking water, but most Cr present in foods is trivalent. The contribution of food group XIV (drinking water) to total Cr exposure was very small (0.25%), and assuming that this was all hexavalent, the exposure level would be 0.001 µg/kg bw/day. This value is well below the TDI of 1.1 μg/kg bw/day for hexavalent Cr and much lower than the mean of 0.04 µg/kg bw/day estimated by the FSCJ under the assumption that the hexavalent Cr concentration in tap water is 2.5 μg/L.

**Hg**

The robust mean of THg exposure was 0.14 μg/kg bw/day, with a range of 0.10-0.18 μg/kg bw/day. The contribution to total exposure level of THg was extremely high in food group X, at 91%, followed by 6.1% in food group I. The contribution by food group XIV was 0.05%. The THg exposure level estimated in the present study was consistent with the estimate (0.15 μg/kg bw/day) reported by the Tokyo metropolitan government in 2020^32^^)^.

The FSCJ established a TDI level of 0.7 μg/kg bw/day for inorganic Hg and 2 μg/kg bw/week (0.28 μg/kg bw/day) for MeHg for pregnant or possibly pregnant women. The Hg in food group XIV is considered to be mostly in the inorganic form. As the overall contribution of group XIV to total exposure level of THg was very small, the exposure represents approximately 50% of the TWI for pregnant women if Hg of food origin is considered mostly MeHg. This suggests that some individuals may be exposed in excess of the indicated amount and reaffirms the need to continue to provide high-risk groups with advice regarding Hg avoidance.

**Ni**

The robust mean of estimated Ni exposure in the present study was 2.8 μg/kg bw/day, with a range of 2.2-3.5 μg/kg bw/day. Food group I contributed the greatest amount (23%) to total Ni exposure. Oguri et al. reported a slightly higher Ni exposure level of 5.6 μg/kg bw/day based on a TDS using an MB approach^18^^)^.

The ratio of the exposure estimate for Ni obtained in the present study to the TDI was 71%. The ratio relative to TDI for Ni may seem high at first consideration. However, as allergic contact dermatitis is used as an index for Ni toxicity, with the exception of Ni-sensitive individuals (i.e., patients with nickel dermatitis), there should be no particular concern over Ni toxicity resulting from oral exposure through the diet.

**Pb**

The robust mean of Pb exposure was 0.15 μg/kg bw/day, with a range of 0.083-0.22 μg/kg bw/day. Only the contribution of food group VIII to total Pb exposure exceeded 20%. The Pb exposure level estimated in the present study was consistent with the estimates of 0.079 μg/kg bw/day (reported value was 4.69 μg/person/day) and 0.095 μg/kg bw/day from previous studies using an MB approach and a DD approach, respectively^17^^,^^19^^)^. In 2020, the Tokyo metropolitan government reported a Pb exposure level of 0.15 μg/kg bw/day^32^^)^. As shown in Fig. 3b, relatively large variabilities were noted depending on region (or, more adequately, the TDS samples prepared), but the nationwide mean annual exposure estimates for Pb in the eight regions (eight TDS samples) fluctuated within certain levels over the 6-year study period.

The FSCJ’s evaluation of Pb concluded that current blood Pb levels in Japan are close to blood Pb concentrations at which epidemiologic studies have suggested some effects, and that efforts should therefore be made to reduce exposure. It is not possible to derive the TDI for Pb from blood Pb concentrations, and there are sources of Pb exposure other than food. The present study did not find a trend of either increasing or decreasing Pb exposure from food, but the greater variability compared with other elements suggests that reductions may be possible if the sources of this variability can be identified.

**Sb**

The robust mean of Sb exposure was 0.022 μg/kg bw/day, with a range of 0.014-0.030 μg/kg bw/day. The ratio of Sb exposure to TDI was 0.4%. This was the lowest ratio calculated in the present study and therefore does not suggest a need for concern.

**Se**

The robust mean of Se exposure was 1.8 μg/kg bw/day, with a range of 1.7-2.0 μg/kg bw/day. The ratio of Se exposure to TWI was 46%. The estimated Se exposure was characterized by a high contribution of animal products to total exposure, with food group X having the greatest amount (35%) and food group XI (meat and eggs) having the second greatest contribution (32%). As Se is also an essential element, it can be concluded that less is not better and Se should not be of concern with regard to harmful effects resulting from excessive exposure.

**U**

The robust mean of U exposure estimated in the present study was 0.021 μg/kg bw /day, with a range of 0.012-0.030 μg/kg bw/day. The ratio of U exposure to TDI was approximately 10%. Food group VIII contributed the greatest amount (65%) to total U exposure. A similar U exposure level (0.03 μg/kg bw/day) was reported by a previous study^18^^)^.

Although U often draws attention for its harmful effects due to radioactivity, the harmful effects of U on the kidneys, which served as the basis for setting the TDI, are the result of chemical toxicity. Although high U concentrations have been reported in mineral water in other countries^33^^)^, the contribution of food group XIV was not high in the present study and therefore should not be of particular concern.

**Co, Mo and Sn**

To date, the FSCJ has not established TDIs for Co, Mo, and Sn. For reference, Co and Mo can be compared with the maximum permissible risk (MPR) levels (MPR 1999/2000) reported by the National Institute for Public Health and the Environment, the Netherlands, in 2001^34^^)^. The MPR levels for Co and Mo were set at 1.4 μg/kg bw/day and 10 μg/kg bw/day, respectively.

The robust mean of Co exposure estimated in the present study was 0.17 μg/kg bw /day, with a range of 0.14-0.20 μg/kg bw/day. The ratio of Co exposure to MPR level was 12%.

The robust mean of Mo exposure estimated in the present study was 4.2 μg/kg bw /day, with a range of 3.6-4.8 μg/kg bw/day. The ratio of Mo exposure to MPR level was 42%.

No appropriate HBGV has been established for Sn. Although further research is needed, there is no reason for concern at this time.

The present study’s estimated exposure levels are generally in agreement with previously reported exposure levels of the relevant elements, indicating that the health risks associated with exposure to these elements have not changed dramatically nationwide in Japan in recent years. In other words, no emerging health risks were identified. However, it must be kept in mind that the daily exposures estimated represent the mean values for the general Japanese population across the entire nation and for all age groups over a certain period. The types and origins of food distributed, as well as human consumption behavior, will change. The results of the present study indicate the importance of continuing to periodically estimate mean exposure levels to elements by TDS to ensure that the health risks to the general Japanese population have not been affected by these changes.
